# Stable CsPbBr_3_ Nanoclusters Feature a Disk-like Shape and a Distorted Orthorhombic Structure

**DOI:** 10.1021/jacs.1c13544

**Published:** 2022-03-08

**Authors:** Baowei Zhang, Davide Altamura, Rocco Caliandro, Cinzia Giannini, Lucheng Peng, Luca De Trizio, Liberato Manna

**Affiliations:** † Nanochemistry Department, Istituto Italiano di Tecnologia (IIT), via Morego 30, 16163 Genova, Italy; ‡ Dipartimento di Chimica e Chimica Industriale, Università degli Studi di Genova, Via Dodecaneso 31, 16146 Genova, Italy; § Istituto di Cristallografia, Consiglio Nazionale delle Ricerche (IC-CNR), Via Amendola 122/O, 70126 Bari, Italy

## Abstract

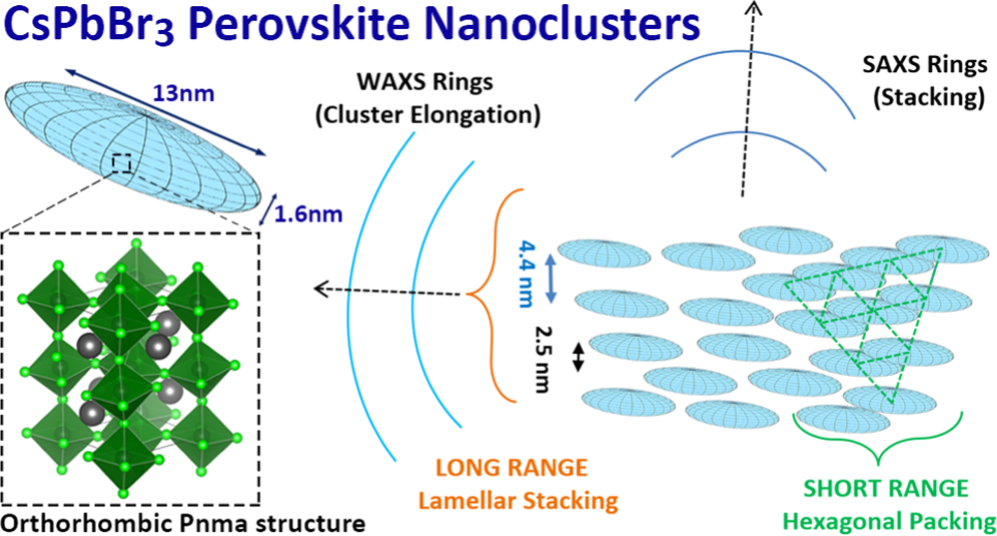

CsPbBr_3_ nanoclusters have been synthesized by several groups and mostly employed as single-source precursors for the synthesis of anisotropic perovskite nanostructures or perovskite-based heterostructures. Yet, a detailed characterization of such clusters is still lacking due to their high instability. In this work, we were able to stabilize CsPbBr_3_ nanoclusters by carefully selecting ad hoc ligands (benzoic acid together with oleylamine) to passivate their surface. The clusters have a narrow absorption peak at 400 nm, a band-edge emission peaked at 410 nm at room temperature, and their composition is identified as CsPbBr_2.3_. Synchrotron X-ray pair distribution function measurements indicate that the clusters exhibit a disk-like shape with a thickness smaller than 2 nm and a diameter of 13 nm, and their crystal structure is a highly distorted orthorhombic CsPbBr_3_. Based on small- and wide-angle X-ray scattering analyses, the clusters tend to form a two-dimensional (2D) hexagonal packing with a short-range order and a lamellar packing with a long-range order.

## Introduction

Nanoclusters (NCLs) are a class of well-defined species that are intermediate in size between molecules and nanocrystals (NCs).^1−7^ They are characterized by an inorganic core, composed of a well-defined number of atoms, passivated by an organic shell made of a stoichiometric amount of surfactants. Having discrete sizes, NCLs can be generally considered as perfectly monodisperse NCs and, indeed, they exhibit narrow optical absorption peaks in the order of homogeneously broadened lines.^8^ In turn, given their small size, they have large surface-to-volume ratios, and their photoluminescence (PL) has a low yield and is often dominated by broad trap emission.^6,9−13^ NCLs have been found to form at the early stages of several colloidal syntheses of NCs. Their isolation and characterization has been often aimed at gaining a better understanding of the nucleation and growth of NCs beyond classical nucleation theory and, consequently, at achieving a higher control over NC synthesis.^2,3,6,8,14^ In this regard, studies on NCLs have revealed that their oriented attachment, continuous growth, or dissolution over time (with the consequent supply of monomers) are key processes involved in the nucleation and growth of NCs.^8,10,15−18^

In addition to their relevance *per se*, NCLs are currently of great interest as they can be used as single-source reagents.^2,6,10,16,19−23^ They represent, in this context, an opportunity to develop new synthetic routes to various types of nanomaterials. The use of NCLs as a single-source precursor can avoid the poorly controllable pyrolytic step in which precursors are converted into monomers (typically occurring at high temperatures), thus enabling greater synthetic control.^19^ This is particularly relevant in the synthesis of nano-heterostructures (or in general in seeded growth approaches) and of those NC systems in which the reactivity of the available precursors cannot be finely tuned, as in the case of III–V semiconductors (e.g., InP and InAs).^15,24^ The interest in such compounds has led, in the last decades, to the isolation and characterization of several NCLs of II–VI and III–V semiconductor materials,^16^ namely, CdS,^3,20,25,26^ CdSe,^17,26,27^ CdTe,^20,26^ ZnS,^20,26^ ZnSe,^20,26^ ZnTe,^18,20^ PbSe,^28^ InP,^2,15,21^ and InAs.^24,29,30^ Only recently, with the emergence of lead halide perovskites, NCLs of APbBr_3_ (A = methylammonium or Cs) materials were discovered.^6,10,13,22,31,32^ CsPbBr_3_ NCLs have been found to form at room temperature in the presence of a high concentration of oleylamine (OLA) and oleic acid (OA), with usually high Pb-to-Cs feed ratios (ranging from 2.5:1 to 6:1).^22,31,32^ CsPbBr_3_ NCLs have been employed as single-source precursors for the synthesis of quantum-confined nanostructures (nanowires, nanoplatelets)^6^ and NCs with complex geometries (i.e., CsPbBr_3_ hexapods)^22^ and heterostructures (i.e., CsPbBr_3_–Pb_4_S_3_Br_2_).^23^ Instead, conventional metal halide precursors (e.g., Cs-oleate and PbBr_2_ or Cs-carbonate, Pb-acetate, and benzoyl bromide)^33,34^ lead to a fast nucleation and growth of CsPbBr_3_ NCs, making it extremely difficult to perform any seeded growth approach or to synthesize heterostructures.^35^

**Scheme 1:**
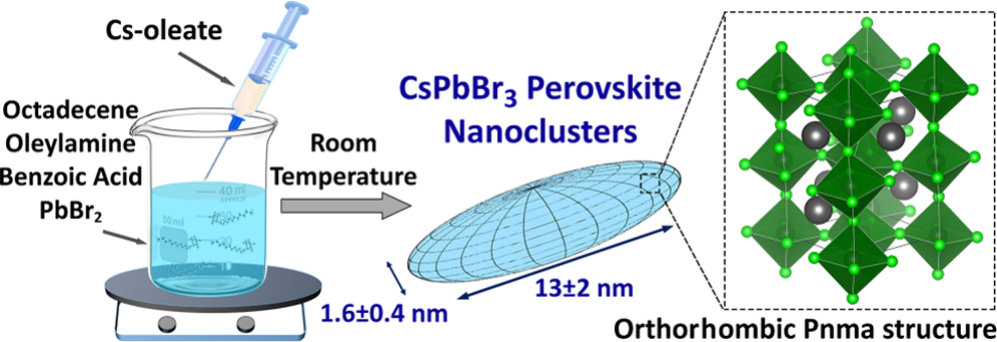
Synthesis, Shape, and Structure of CsPbBr_3_ NCLs

Despite their importance in the synthesis of perovskite-based nanostructures, stable CsPbBr_3_ NCLs have never been successfully prepared and, consequently, characterized in depth.^32^ In fact, the CsPbBr_3_ NCLs reported so far typically grow larger already at room temperature over a timespan of a few minutes and they cannot be purified with the use of polar solvents.^6,13,31^ With the aim of increasing the stability of such materials, different strategies have been pursued: Xu et al. used either aluminum nitrate nonahydrate as a coordination complex together with oleylamine and oleic acid^32^ or a combination of benzoic acid (BA) and benzylamine.^36^ In both cases, no stable and/or pure CsPbBr_3_ NCLs could be isolated. To circumvent such issues, we have developed here a new strategy based on oleylamine and benzoic acid ligands (which have been successfully employed to prepare the more conventional CdSe, CdS, and InP semiconductor NCLs^4,11,37,38^) to synthesize stable CsPbBr_3_ NCLs (Scheme 1). These nanostructures are characterized by a sharp excitonic absorption peak at 399 nm and a room-temperature photoluminescence (PL) emission featuring a sharp peak at 410 nm and a long tail extending up to 600 nm. While the peak at 410 nm can be ascribed to band-edge emission, the broad tail is attributed to surface trap states, similar to that observed in NCLs of II–VI or III–V compounds.^12,39^ Our clusters can be washed with polar solvents, and they are found to be stable over one week at room temperature both in concentrated hexane solutions (>50 mg/mL) and in solid form (under N_2_). Such stability allowed us to perform a detailed chemical and structural analysis by combining different techniques, comprising, *inter alia*, pair distribution function (PDF) as well as small- and wide-angle X-ray scattering (SAXS, WAXS). The composition of NCLs was estimated to be CsPbBr_2.3_, and their crystal structure was identified by PDF data as orthorhombic CsPbBr_3_, although highly distorted. Convergent evidence from PDF, SAXS, and WAXS analyses on solid samples indicated that the clusters have an anisotropic, disk-like shape, with a 1.6 ± 0.4 nm thickness and 13 ± 2 nm diameter (Scheme 1). Based on SAXS, the clusters form assemblies having 4.4 and 2.5 nm periodicities, indicating a hexagonal short-range order and a lamellar long-range order. We hypothesize that such a mesophase could be the reason for the enhanced stability of our NCLs, similar to that reported for CdS NCLs.^4,40^

Our work not only provides a way to produce stable NCLs to be employed on demand as single-source precursors for the synthesis of perovskite-based complex structures, but also sheds light onto perovskite NCLs by revealing that they are not similar to classical NCLs, which have more isotropic shapes and sizes in the order of 1–3 nm. Instead, the present NCLs are confined platelets with larger lateral dimensionality.

## Experimental Section

### Chemicals

1-Octadecene (ODE, tech, 90%), oleic acid (OA, tech, 90%), benzoic acid (BA, 99%), oleylamine (OLA, tech, 70%), lead(II) bromide (PbBr_2_, 98%), and cesium carbonate (Cs_2_CO_3_, 99%) were purchased from Sigma-Aldrich. The reagents were used as received without any further experimental purification. ODE and OLA were degassed before use.

### Synthesis of the Cesium Oleate Precursor

In a typical synthesis, Cs_2_CO_3_ (0.652 g, 2 mmol) and OA (6.5 mL, 20.4 mmol) were loaded into a 50 mL three-neck flask degassed for 2 h at 100 °C until the solution turned clear.

### Synthesis of CsPbBr_3_ NCLs

PbBr_2_ (71 mg) was mixed with 100 mg of benzoic acid, 0.5 mL of oleylamine, and 4.5 mL of ODE in N_2_-filled 20 mL glass vials. The mixture was heated at 150 °C for 5 min to obtain a clear solution. After cooling back to room temperature, 150 μL of Cs-oleate stock solution was injected into the clear solution, and the resulting mixture was kept under stirring at room temperature (25 °C). After about 3 h, 20 mL of ethyl acetate was added and the resulting mixture was centrifuged at 6000 rpm for 10 min, the supernatant was discarded, and the precipitate was redispersed in 1 mL of anhydrous hexane for storage.

### Elemental Analysis

Energy-dispersive X-ray spectroscopy (EDS) was performed on a JEOL JSM-7500FA SEM-Analytical field-emission scanning electron microscopy (SEM) with an Oxford X-Max 80 system equipped with an 80 mm^2^ silicon drift detector (SDD). X-ray photoelectron spectroscopy (XPS) was performed on a Kratos Axis UltraDLD spectrometer, equipped with a monochromatic Al Kα source, which was operated at 20 mA and 15 kV. Spectra were charge corrected to the main line of the carbon 1s spectrum (adventitious carbon) set to 284.8 eV. Spectra were analyzed using CasaXPS software (version 2.3.24).

### UV–Vis Absorption and Photoluminescence (PL)

The UV–visible absorption spectra were recorded using a Varian Cary 300 UV–vis absorption spectrophotometer. The PL spectra were collected by a Varian Cary Eclipse fluorescence spectrophotometer.

### Pair Distribution Function (PDF)

PDF measurements were performed at the National Synchrotron Light Source (NSLS-II) of the Brookhaven National Laboratory. The 28ID-2 beamline was used with a primary X-ray beam of 67.17 keV (0.1846 Å) energy and 0.5 mm × 0.5 mm spot size. A PerkinElmer X-ray diffraction (XRD) 1621 digital imaging detector (2048 × 2048 pixels of 200 μm × 200 μm size) orthogonal to the beam was put 242 mm downstream the sample to optimize PDF measurements. Nickel was measured as a standard reference material to calibrate the wavelength and the detector position/orientation. Fresh samples were enclosed into small bags of ultralene (Figure S1) and sealed to preserve cluster properties prior and during X-ray measurements. An empty bag was measured for background estimation. X-ray measurements were performed at 270 K with no filters and without spinning the sample. Diffraction images were azimuthally integrated and converted into intensity profiles versus 2ϑ and versus momentum transfer (*Q*) using the FIT2D program.^41^ PDF profiles were calculated up to interatomic distances *r* of 40 Å from *Q* profiles by the program PDFGetX3.^42^ The parameters for PDF calculation (background subtraction scale factor, minimum and maximum values of *Q*, degree of data-correction polynomial) were optimized to reduce termination effects and to enhance the signal-to-noise ratio. The *Q*_max_ parameter was set to 21.2 Å^–1^.

The PDF profile was refined using a python script based on the DiffPy-CMI library.^43^ The fits were executed for interatomic distances above 2.0 Å to avoid finite-size artifacts in the low *r* range with a step size of 0.05 Å. The fitting model was defined as the convolution of the PDF contribution due to a bulk crystal structure and that due to the nanocrystal shape. The model parameters were refined separately, i.e., by keeping all of the others constant, with the following order: scale factor, nanocrystal shape parameters (radius in the case of spherical shape or polar/equatorial radii in the case of spheroidal shape), peak shape parameters (*Q*_broad_, that is the peak broadening from increased intensity noise at high *Q*, and delta1, that is the coefficient for 1/*r* contribution to the peak sharpening), atomic displacement parameters, lattice parameters, atomic position parameters, and crystallographic occupancy.

In the first stage, a simplified fit procedure restricted to *r* < 13 Å was used to screen the 22 CsPbBr crystal structures present in the ICSD database. Ten refinement cycles were performed using a spherical nanocrystal shape and isotropic displacement factors. The atomic positions and crystallographic occupancies were kept fixed. In the second stage, the full PDF range was exploited to carry out a more elaborate refinement of the selected crystal phase. Fifty refinement cycles were performed, where the atomic displacement parameters were kept isotropic in the first 25 cycles, and then set to anisotropic in the last 25 cycles. A spheroidal NC shape was used, and the displacement parameters of each element and the atomic parameters of each atom were refined separately.

### Small- and Wide-Angle X-ray Scattering (SAXS, WAXS)

SAXS and WAXS data were collected at the XMI Lab^44^ using a Fr^–^E+ superbright microsource (Cu Kα) coupled to a SMAX3000 camera (Rigaku). SAXS data were collected by a multiwire Triton detector, at 1.140 m sample-to-detector distance (SDD); Kapton windows were inserted upstream and downstream the sample to maintain it at atmospheric pressure (flight tube at about 10^–1^ mbar vacuum pressure). SAXS data from solutions were obtained by filling a glass capillary with 50 mg/mL solution of clusters in hexane, and a capillary with the only hexane as a buffer, for solvent scattering subtraction. One-dimensional (1D)-folded SAXS profiles were fitted using the ATSAS suite.^45^ SAXS microscopies were conducted in scanning mode on solid samples, inserted in ultralene bags with a 0.2 mm step size, and processed by the in-house developed SUNBIM package,^46^ exploiting the multimodal imaging approach.^47^ WAXS data were collected at selected positions on the sample (simultaneously to SAXS data acquisition), by inserting an Image Plate (IP) detector at a 28 mm SDD downstream the sample and using a RAXIA scanner for off-line readout.

### Dynamic Light Scattering (DLS)

The Malvern Zetasizer (Nano Series, Nano ZS) instrument was used to determine the hydrodiameter of the NCs. Three measurements with 10–20 acquisitions were taken for each sample.

### High-Angle Annular Dark-Field Scanning Transmission Electron Microscopy (HAADF-STEM)

HAADF-STEM images were acquired using an image-Cs-corrected JEM-2200FS microscope, operating at 200 kV. The specimen was prepared by drop casting the sample suspended in octane onto an ultrathin carbon/holey carbon/Cu grid.

## Results and Discussion

### Synthesis of CsPbBr_3_ NCLs

**Figure 1:**
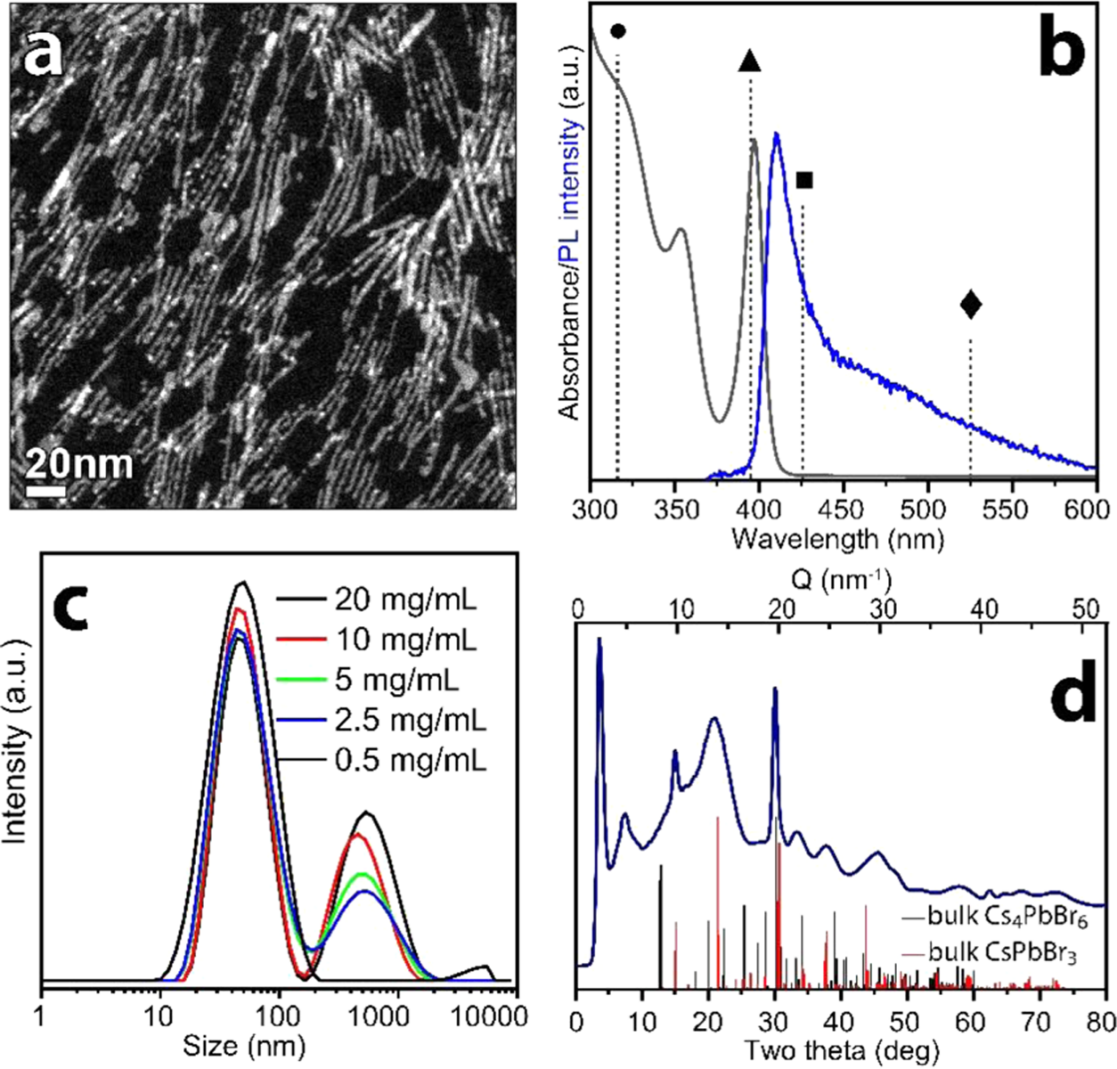
(a) HAADF-STEM image and (b) absorption and photoluminescence spectra of purified CsPbBr_3_ NCLs. The vertical dashed lines represent the spectral positions of the absorption peaks related to the following species, if they were present in the sample: zero-dimensional (0D) Cs_4_PbBr_6_ (●),^53^ 1 monolayer (oleylammonium)_2_PbBr_4_ nanosheets (▲),^50−52^ CsPbBr_3_ nanoplatelets with 2 monolayers thickness (■),^48^ and bulk CsPbBr_3_ (⧫). (c) DLS analysis of CsPbBr_3_ NCLs dispersions in hexane at different concentrations. (d) X-ray powder diffraction (XPD) pattern of CsPbBr_3_ NCLs measured at the NSLS-II synchrotron source. 2θ values are those for Cu Kα radiation for the sake of comparison. The bulk reflections of orthorhombic CsPbBr_3_ (ICSD 98751) and Cs_4_PbBr_6_ (ICSD 162158) structures are reported as red and black bars, respectively.

The CsPbBr_3_ NCLs reported in this work were synthesized following the procedure reported by Peng et al.^6^ and by substituting oleic acid with benzoic acid. The obtained crude solution was optically transparent and colorless, and the product could be precipitated as a white solid by the addition of ethyl acetate (i.e., antisolvent) followed by centrifugation after 2–3 min. To define the elemental composition of NCLs, we performed XPS and SEM-EDS analyses (Figures S2, S3, Tables S2, and S3). Both analyses indicated that NCLs have a CsPbBr_2.3_ composition with oleylammonium and carboxylate ions (the latter identified via XPS analysis, see Table S3) ensuring charge balance. Unfortunately, a precise quantification of the two surfactants could not be achieved as the clusters degraded if subjected to multiple washing steps (required to get rid of excess unbound ligands). The CsPbBr_3_ NCLs were characterized by a sharp excitonic absorption peak at ∼399 nm along with two additional absorption peaks at higher energies (353 and 318 nm), the same as the reported NCLs by Peng et al. and by other groups,^6,22,23,31^ and representing the fine exciton structure (Figure 1b). The PL emission of NCLs was composed of a relatively narrow peak at 410 nm and a broad tail extending up to 600 nm (Figure 1b), which have been tentatively ascribed to band gap and surface trap emission, respectively. Such optical features indicate that our NCLs are in the strong confinement regime since their absorption peak falls: (i) at higher energies with respect to that of bulk CsPbBr_3_ and of 2 monolayer (ML) thick CsPbBr_3_ nanoplatelets (∼100 nm diameter);^48^ (ii) at lower energies with respect to that of Cs_4_PbBr_6_ structures in which all of the PbBr_6_ octahedra are disconnected from each other.^49^

Indeed, our NCLs featured optical properties that are analogous to those of 1ML micron-sized nanosheets with the formula (oleylammonium)_2_PbBr_4_.^50−52^ These nanosheets do not contain Cs^+^ cations, and hence the [PbBr_6_]^4–^ octahedra form a two-dimensional (2D) network limited to 1ML and are stabilized by oleylammonium ions. Such nanosheets are characterized by an excitonic absorption peak located at ∼398 nm, similar to the present work, but they lack a fine excitonic structure (i.e., they do not feature an absorption peak at 350 nm),^50−52^ as instead seen in our samples. The PL emission of such nanosheets is also similar to that of our NCLs, which have a PL peak located at ∼403 nm and a long tail toward lower energies.^51,52^ These considerations indicate that the confinement in our NCLs is similar to that of 1ML organic–inorganic lead bromide nanosheets.

HAADF-STEM analysis was performed to reveal the morphology of the NCLs, which appeared as thin (∼2 nm) elongated nanostructures (Figure 1a). According to DLS measurements, the cluster dispersions in hexane were composed of objects having large hydrodynamic diameters (in the range of 500 and 50 nm), indicating that NCLs formed large assemblies (Figure 1c). Upon dilution, such assemblies gradually disappeared, and, below the concentration of 0.5 mg/mL, the NCL solution became light green in color, with absorption at 420 nm, indicating the formation of CsPbBr_3_ NCs, as shown in Figure S4. Overall, these findings suggest that the concentration of NCLs in solution influences their aggregation, which in turn plays a key role in their stabilization.

The cluster samples were found to be stable up to 2 weeks when stored as concentrated dispersions in hexane (50 mg/mL) at room temperature, after which an absorption peak at 420 nm was observed, signifying the formation of three-dimensional (3D) perovskite nanostructures (Figure S5). The validated stability of NCLs allowed us to perform powder XRD (XPD) measurements and synchrotron experiments, which were optimized to carry out PDF, the latter particularly suited to investigate the local structure of nanomaterials (Figure 1d).^54^ It can be noted that a portion of the XRD reflections of NCLs matched with those of the orthorhombic CsPbBr_3_ phase (Figure 1d) apart from the peaks at 2θ < 10° that could not be indexed with any known Cs–Pb–Br crystal phase, thus being tentatively ascribed to a supramolecular arrangement of NCLs.

**Figure 2:**
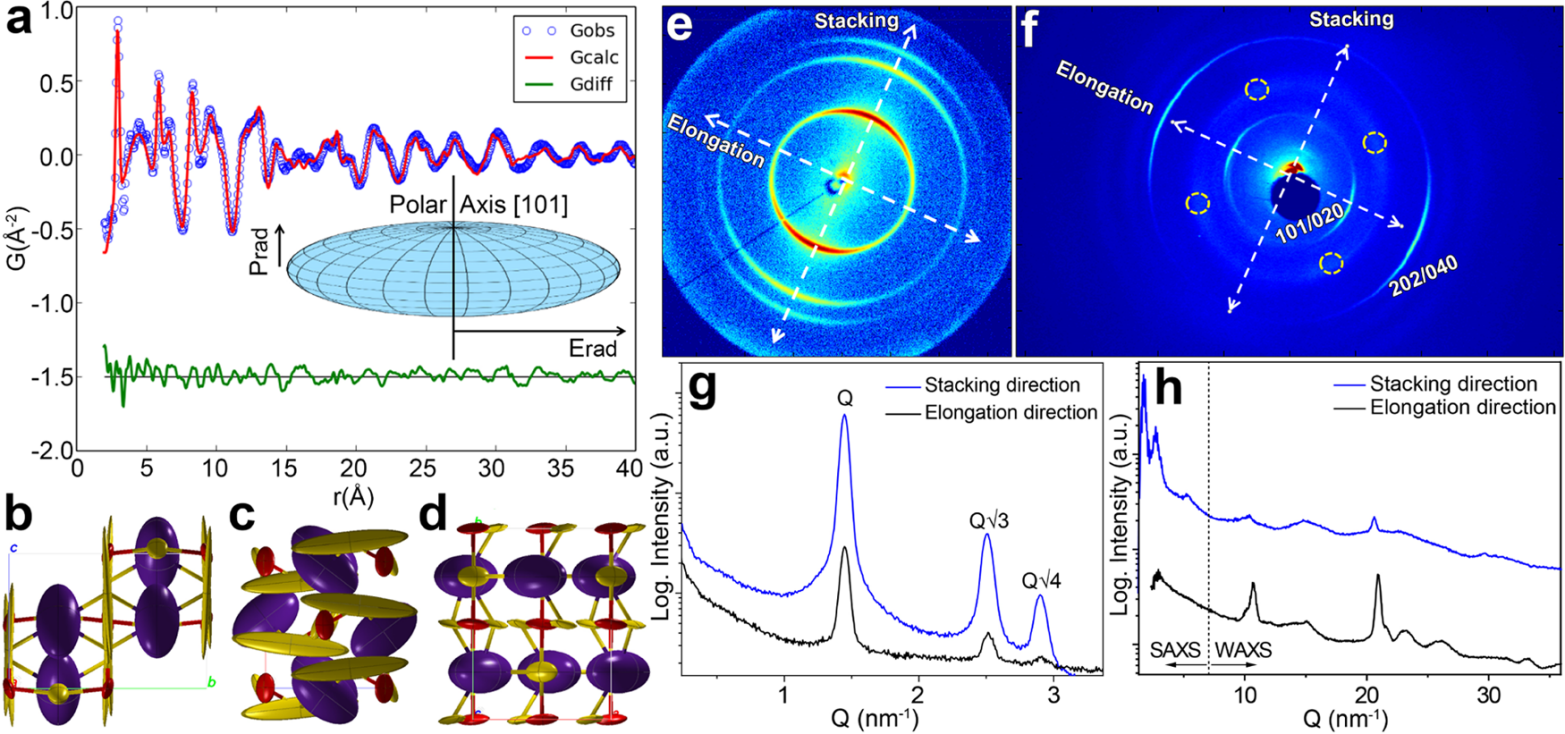
PDF fit (a) and refined crystal structure viewed normal to the *a*, *b*, *c* axes (panels b, c, d panels, respectively) of the orthorhombic CsPbBr_3_ phase. Pb, Cs, and Br ions are shown in red, violet, and yellow, respectively. Thermal ellipsoids at 50% probability level represent their atomic anisotropic displacement factors. Inner picture of (a): schematic view of the NCL shape, based on PDF refinement and microstructural analysis of the XPD profile. *E*_rad_ and *P*_rad_ are respectively the equatorial and polar radii parameters (Table S1). (e) SAXS and (f) WAXS 2D patterns simultaneously collected at the XMI Lab, at a selected sample position; (g) 1D-folded SAXS pattern from the line cuts indicated by the white arrows in panel (e); (h) 1D-folded WAXS patterns from the line cuts indicated by the white arrows in panel (f).

The local structure of NCLs was investigated by calculating the PDF profile from the measured XPD pattern shown in Figure 1d. To this aim, all existing crystal phases in the ISCD database were screened against PDF data using a fitting procedure that was restricted to interatomic distances <13 Å, both to avoid interference with the supramolecular arrangement and to increase the sensitivity of the identification. The results indicate that the best match with the PDF profile could be achieved with the orthorhombic *Pnma* crystal structure (Figure S6).^55,56^ A more elaborate refinement procedure was then carried out on the selected orthorhombic crystal phase to retrieve information about the anisotropic atomic motion and NCL shape. The best fit, reported in Figure 2a, was achieved when setting the shape of the NCLs as an oblate spheroid having equatorial and polar diameter of 13 ± 2 and 1.6 ± 0.4 nm, respectively (Figure 2a and Table S1). In such a model, Cs^+^ cations are characterized by an anisotropic motion along the [101] and [1̅01] directions (Figure 2b–d, violet spheroids), while the Br^–^ anions in general positions fluctuate along the [001] direction (Figure 2b–d, longer red disks).

To further confirm these results, we also performed a microstructural analysis by whole profile fitting (WPF) of the synchrotron XPD profile (see Figures S7 and S8), which returned an estimate of the average size for crystallite domains of 10.7 nm from the (020) reflection and 2.3 nm from the (101) reflection. Such dimensions agree with the PDF shape determination if the polar axis of the spheroid is directed along the [101] direction (n.b. the size values returned by the WPF should not be considered strictly quantitative: the low goodness of fit indicates the low efficiency of this approach, likely due to the high preferred orientation and anisotropy of NCLs). An additional confirmation of the shape anisotropy of the clusters was obtained by SAXS measurements of 50 mg/mL solutions of NCLs in hexane (such measurement is not sensitive to the atomic structure of the clusters, but only to their nanoscale morphology). The SAXS fitting indicated an asymmetric pair distribution function [*p*(*r*)], peaked at about 2.5 nm, featuring a maximum interatomic distance (i.e., cluster long axis) of about 13 nm and a radius of gyration of 4 nm, which is consistent with an object having a disk-like shape and dimensions as those estimated by PDF (see also Figure S9 and discussion therein).

**Scheme 2:**
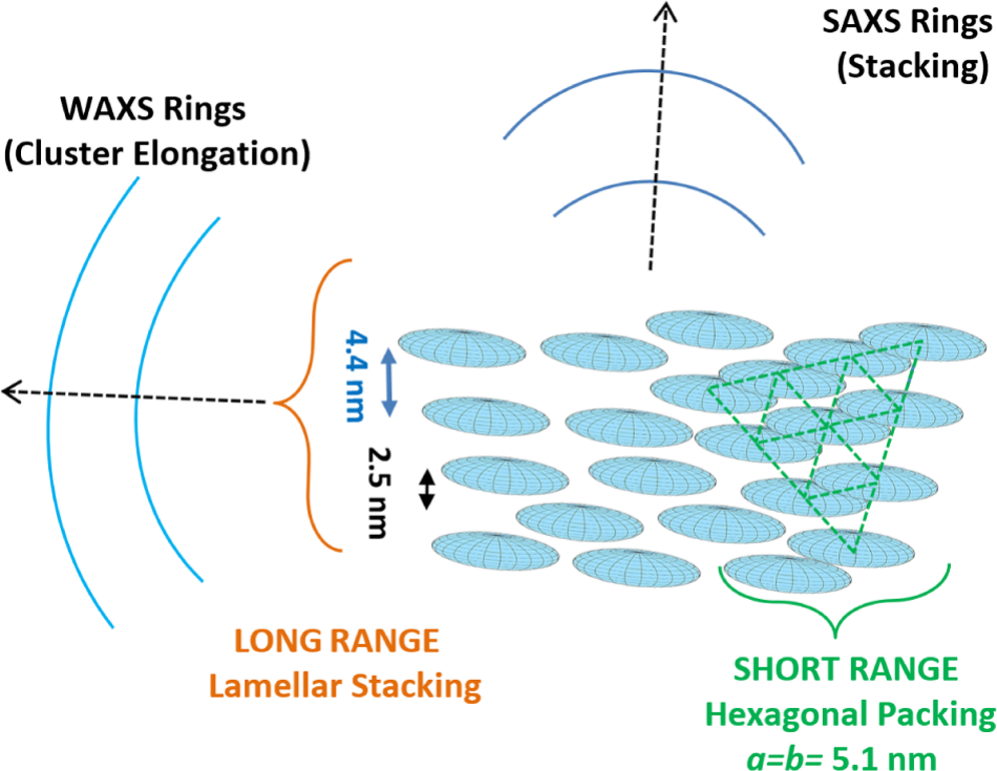
NCL Arrangement According to SAXS and WAXS Patterns

To further investigate NCLs packing, we performed combined SAXS and WAXS measurements in the solid state. The SAXS signal (Figure 2e,g) explains the XRD peaks at small angles in terms of ordered stacking of NCLs in a superstructure, while the WAXS signal (Figure 2f,h) is consistent with XPD measurements and reveals an elongation direction of NCLs orthogonal to the stacking direction (compare Figure 2e,f, and Scheme 2). Indeed, based on the whole profile fit in Figure S7, the most intense partial diffraction rings appearing in Figure 2f could be clearly identified as the (101)/(020) and (202)/(040) reflections relative to the orthorhombic CsPbBr_3_ crystal phase. Moreover, the simultaneous collection of SAXS and WAXS patterns at the same sample position allowed us to reveal the coherent orientation of NCLs in the assembly: (i) the stacking direction can be extracted by the partial rings of the SAXS pattern (Figure 2e); (ii) NCLs orientation is defined by the main WAXS partial rings (Figure 2f) perpendicular to the stacking direction. It is worth to note that the partial rings in the SAXS pattern (Figure 2e) fulfill the *Q*-positions (Figure 2g) expected for a hexagonal packing: *Q*, *Q*√3, 2*Q* (i.e., (100), (110), (200) reflections, respectively).^40^ However, an additional peak expected at *Q*√7 is missing, and it is apparently replaced by a broad peak appearing around 5.2 nm^–1^ (2θ = 7.4°) in the WAXS pattern (Figure 2h). At the same time, no peaks are detected around 0.6 nm^–1^ (which would be expected in the case of a periodic arrangement along the equatorial plane of ∼10 nm clusters). Such results have been confirmed at any sample position by collecting scanning SAXS microscopies (Figure S10). As a consequence, a 2D hexagonal symmetry can be expected with a short-range order, with unit cell parameter *a* = 4.4/cos 30° = 5.1 nm, while a lamellar stacking is observed on a larger length scale, with periodicities of 4.4 and 2.5 nm (Scheme 2). Such two periodicities could refer to vertically aligned or shifted (close-packed) clusters. The lack of long-range order in three dimensions can be likely ascribed to the hindrance in the equatorial plane of the clusters (Figures 2a and S8), preventing them from getting close enough to form a regular 3D assembly.

Overall, we can state that, on average, NCLs could be described as 1 monolayer-thick platelets. Yet, their peculiar morphology, crystal structure, and surface chemistry make them behave differently from previously reported perovskite nanoplatelets. For example, both ultrathin organic–inorganic (oleylammonium)_2_PbBr_4_ nanosheets (1 monolayer thick)^50−52^ and few-monolayers-thick CsPbBr_3_ platelets^57^ have been shown in previous works to assemble in layered structures with high preferred orientation. Such orientation was evident from the XRD patterns, which featured several equally spaced peaks in the small-angle range and also intense peaks in the wide-angle range that were related to the in-plane crystalline order. Also, the previously reported nanoplatelets featured a cubic CsPbBr_3_ structure.^51,57^ Conversely, NCLs in the present work exhibit a distorted orthorhombic CsPbBr_3_ phase and they are not able to form layered structures with long-range order. Instead, they assemble based on their particular shape and size, as discussed in this work. For these reasons, NCLs presented here are considered different from simple nanoplatelets.

## Conclusions

In summary, our analyses indicate that CsPbBr_3_ NCLs synthesized in this work exhibit: (i) a CsPbBr_2.3_ stoichiometry; (ii) a highly distorted orthorhombic *Pnma* crystal structure; (iii) an oblate spheroid shape having an equatorial length of 13 ± 2 nm and polar diameters of 1.6 ± 0.4 nm; and (iv) 2D hexagonal packing with short-range order and a lamellar packing with long-range order. Such features, therefore, explain the optical spectra of NCLs, where a strong quantum confinement is expected along the polar direction and a weak confinement along the equatorial direction. Our results shed light on CsPbBr_3_ NCLs by disclosing their structure, shape, and assembly geometry. We believe that the clusters reported here, thanks to their improved stability, will be employed as stable single-source precursors in the synthesis of new perovskite nanocrystals and perovskite-based nano-heterostructures.

## Supporting Information Available

Refinement parameters derived from the PDF fit, SEM-EDS, and XPS analysis, abs curve vs time of NCLs in different concentrations, crystal phase identification, whole profile fitting of the synchrotron XRD pattern, fit of the 1D-folded SAXS, and orientation/intensity SAXS microscopies (PDF)
