# Supplementary material for: Stable CsPbBr3 Nanoclusters Feature a Disk-like Shape and a Distorted Orthorhombic Structure
Source: J Am Chem Soc. 2022 Mar 8;144(11):5059–66. doi: 10.1021/jacs.1c13544 (PMC8949727; doi:10.1021/jacs.1c13544)
Supplement: Supplementary file 1 — ja1c13544_si_001.pdf [file ja1c13544_si_001.pdf]

Supporting Information for:

Stable $CsPbBr_3$ Nanoclusters feature a Disk-like Shape and a Distorted Orthorhombic Structure

Baowei Zhang,[a,b] Davide Altamura,[c]* Rocco Caliandro,[c] Cinzia Giannini,[c] Lucheng Peng,[a] Luca De Trizio,[a]* Liberato Manna[a]*

[a] Nanochemistry Department, Istituto Italiano di Tecnologia (IIT), via Morego 30, 16163 Genova, Italy.

[b] Dipartimento di Chimica e Chimica Industriale, Università degli Studi di Genova, Via Dodecaneso 31, 16146 Genova, Italy.

[c] Istituto di Cristallografia, Consiglio Nazionale delle Ricerche (IC-CNR), Via Amendola 122/O, 70126-Bari, Italy

Corresponding Authors

davide.altamura@ic.cnr.it
luca.detrizio@iit.it
liberato.manna@iit.it

Table S1. Refinement parameters derived from the PDF fit of the orthorhombic $CsPbBr_3$.crystal phase. R_w is the weighted agreement factor between observed and calculated PDF; Delta1 is the coefficient for 1/*r* contribution to the peak sharpening*;* Erad and Prad are the equatorial and polar radii of the spheroidal shape (Fig. 2a in the main text) assumed for the nanocluster; *a*,*b*,*c* are the length of the unit cell axes.

Rw	0.257
Delta1	2.5±0.5
Erad (Å)	64±9
Prad (Å)	8±2
a,b,c (Å)	8.5±0.2, 11.8±0.1, 8.0±0.2

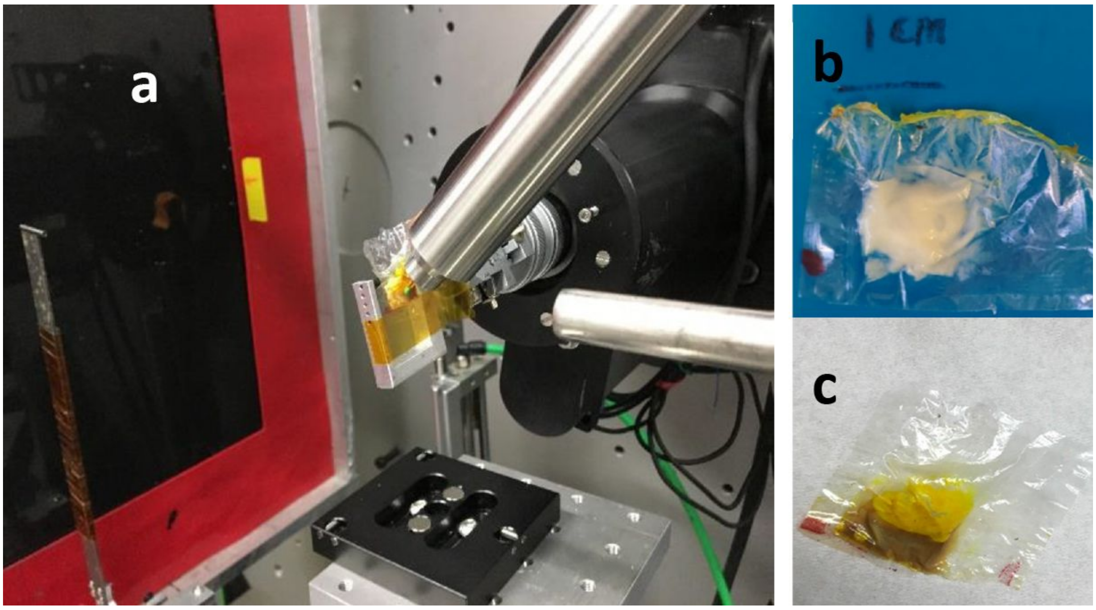

Figure S1. Experimental setup for PDF measurements, with the nanocluster sample put in an ultralene bag (a) and (b) fresh NCLs sample for test; (c) NCLs sample after heating.

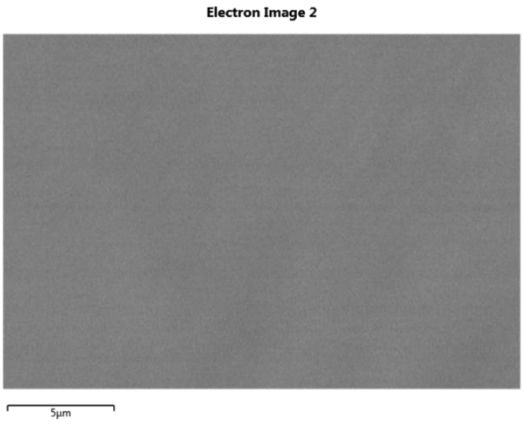

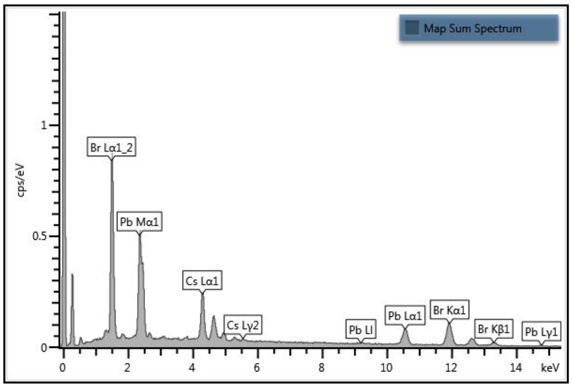

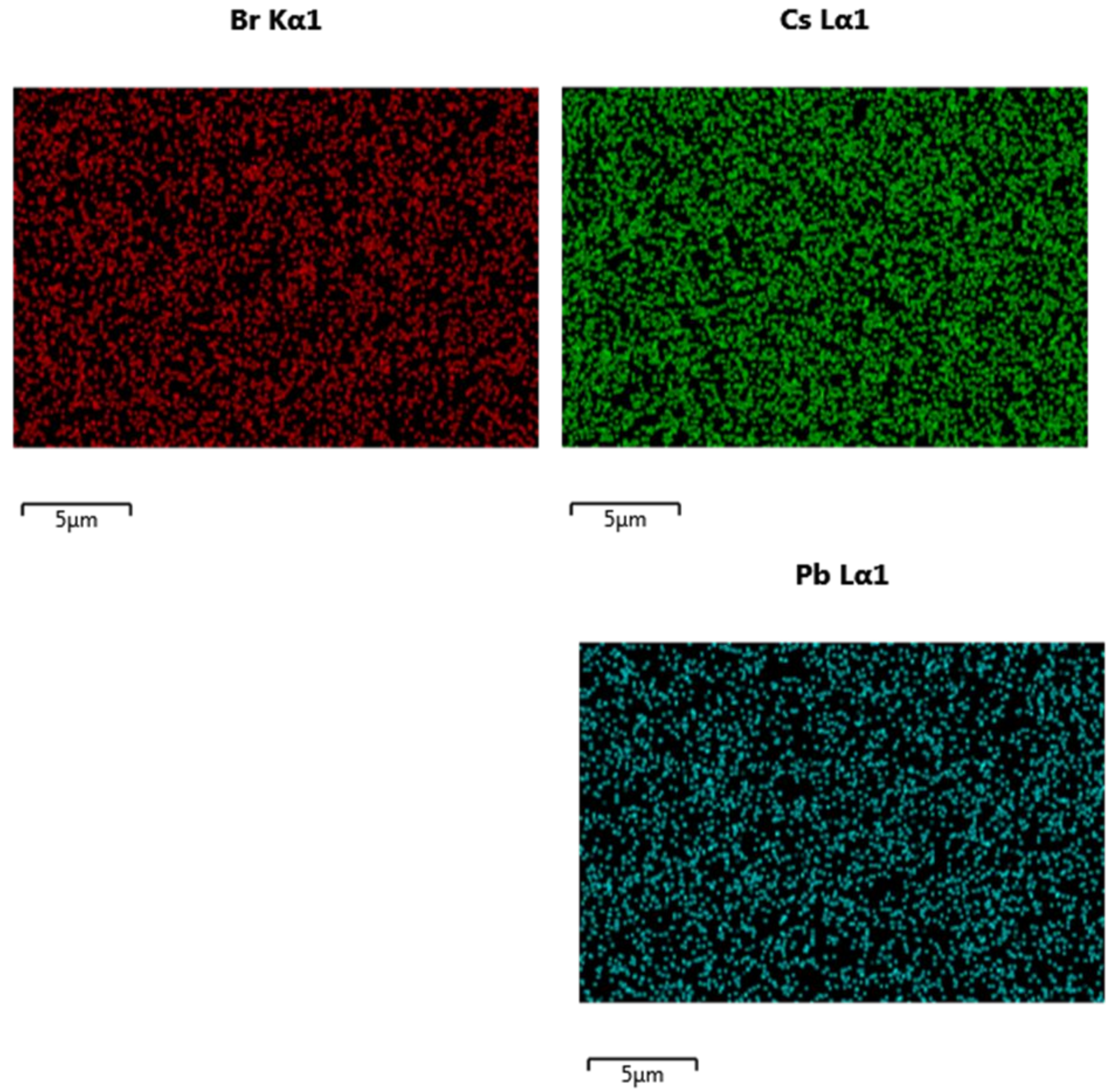

Figure S2. SEM-EDS mapping of the purified $CsPbBr_3$ nanoclusters sample.

Table S2. SEM-EDS analysis of the purified $CsPbBr_3$ nanoclusters sample.

Map Sum Spectrum	Line Type	Wt%	Wt% Sigma	Atomic %
**Br**	K series	35.10	0.75	53.56
**Cs**	L series	25.09	0.53	23.02
**Pb**	L series	39.80	0.84	23.42
**Total**		100.00		100.00

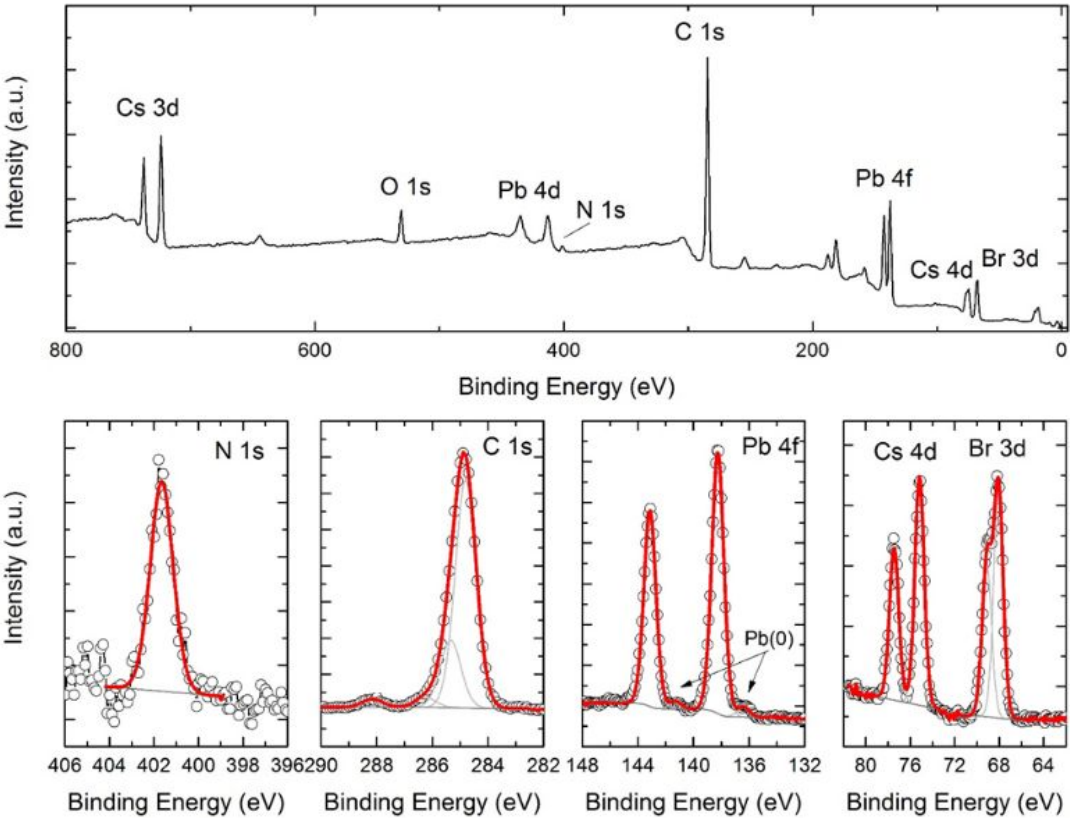

Figure S3. XPS spectra of the purified $CsPbBr_3$ nanoclusters sample.

Table S3. XPS result of the purified $CsPbBr_3$ nanoclusters sample. The COO^- content was estimated from the area of the C 1s component centered at 288.1±0.2 eV, while that of NH_3^+ from the N 1s peak at 401.6 eV, in agreement with what reported by Gonella et al., *J. Phys. Chem.* B 2005, 109, 38, 18003–18009.

Element	Mol ratio (%)
**Cs**	16.3
**Pb**	17.1
**Br**	39.9
**COO^-**	16.0
**NH_3^+**	10.7

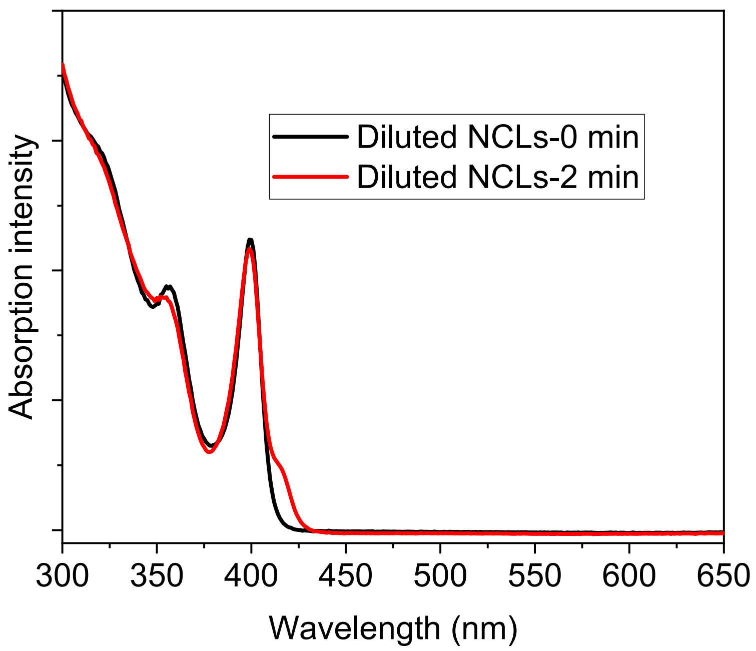

Figure S4. Optical absorption spectra of a diluted dispersion of NCLs in hexane (0 min) and their transformation into $CsPbBr_3$ after 2 min of ageing under ambient atmosphere .

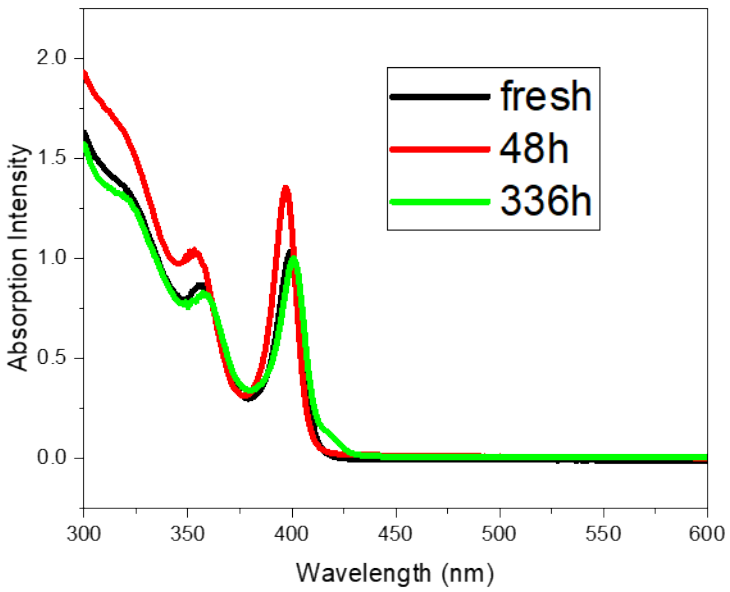

Figure S5. Time evolution of the optical absorption spectrum of concentrated (50 mg/ml) $CsPbBr_3$ nanoclusters dispersed in hexane.

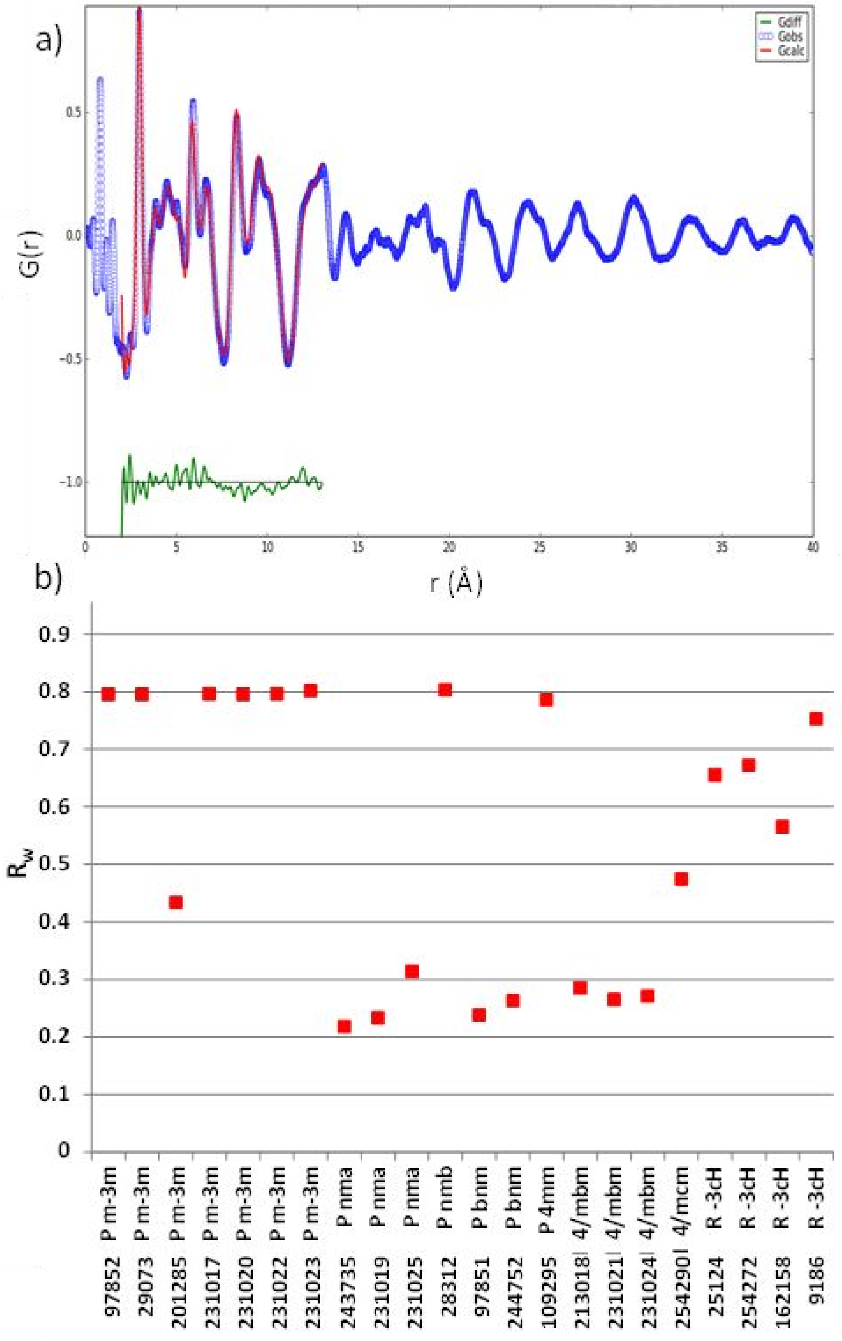

Figure S6. (a) PDF profile of the $CsPbBr_3$ NCLs fitted in a restricted range by the crystal phase Pnma. Experimental (blue dots), calculated (red line) and difference (green line) PDF values are shown. (b) Results of the crystal phase identification procedure. Weighted agreement factor (R_w) between observed and calculated PDF, obtained by refining 22 Cs-Pb-Br crystal phases present inthe ISCD, identified by their space group and entry code. The Pnma crystal structures n.231019[1] and 243735[2], which minimize R_w and are in fact very similar, best fit the PDF profile.

---

[1] Patrick Cottingham and Richard L. Brutchey : Chem. Mater. 2018, 30, 6711−6716.

[2] Matthew R. Linaburg, Eric T. McClure, Jackson D. Majher, and Patrick M. Woodward Chem. Mater. 2017, 29, 8, 3507–3514.

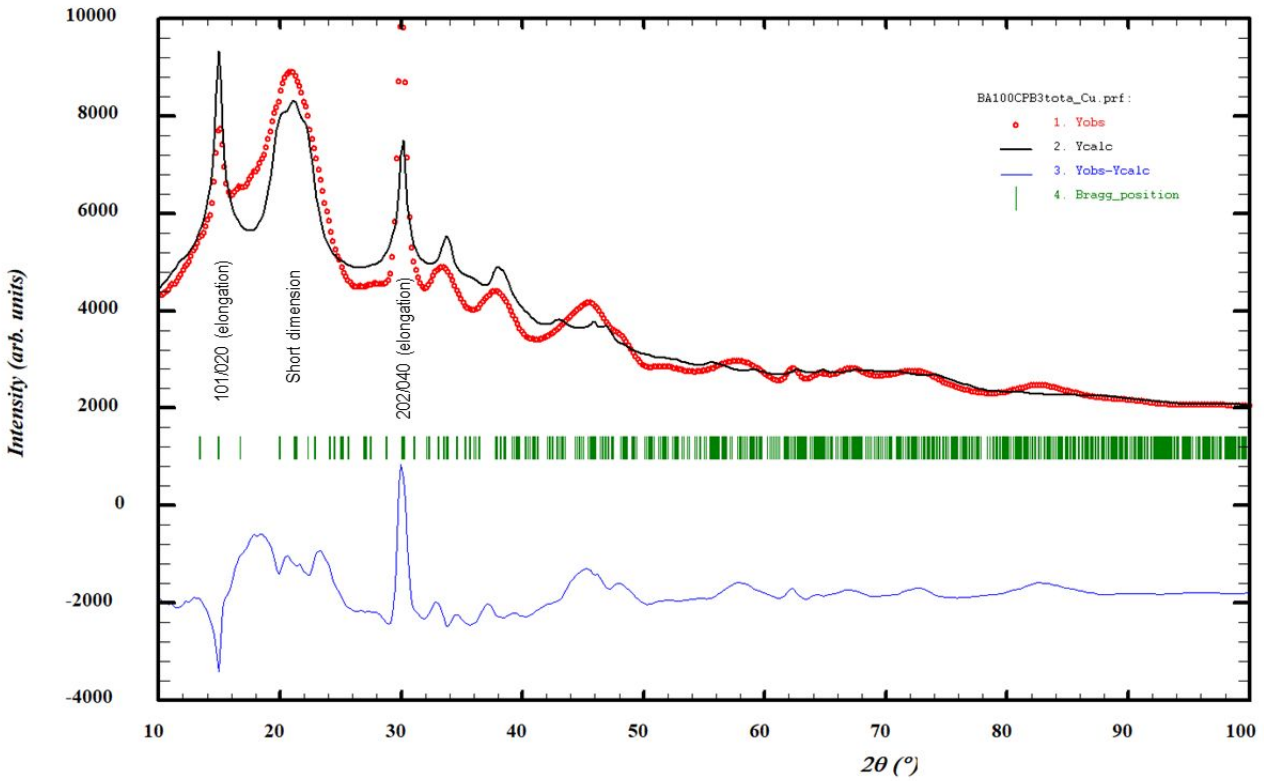

Figure S7. Whole profile fitting of the synchrotron XRD pattern (reported in Figure 1d of the main text), showing the anisotropic shape of clusters, based on the significantly different FWHM of the diffraction peaks.

Whole profile fitting in Figure S5 was performed by using the program FullProf[3]. Although a perfect fit could not be achieved, probably due to high preferred orientation and anisotropy of the nanoclusters, a size difference of at least a factor 4 was found along the two main perpendicular directions (i.e. 2.3 and 10.7 nm for the 101 and 020 reflections, respectively). Based on the FWHM of all peaks in the calculated profile, the program returned an overall disk-like shape of the single cluster, as reported in Figure S6.

[3] Juan Rodríguez-Carvajal Recent advances in magnetic structure determination by neutron powder diffraction Physica B: Condensed Matter 192, 1993, 55-69.

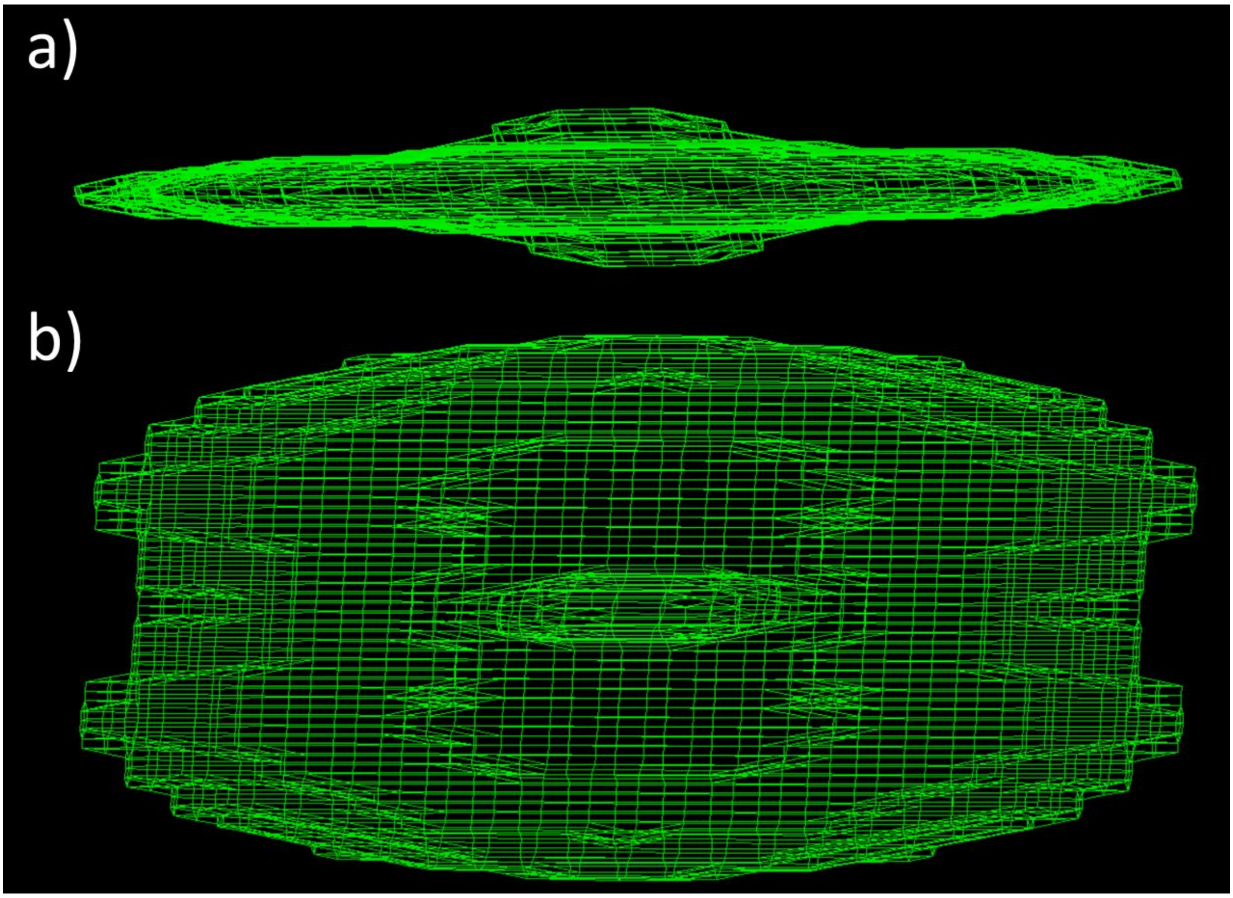

Figure S8. Overall the clusters shape resulting from the whole profile fit. View parallel (a) and perpendicular (b) to the equatorial plane.

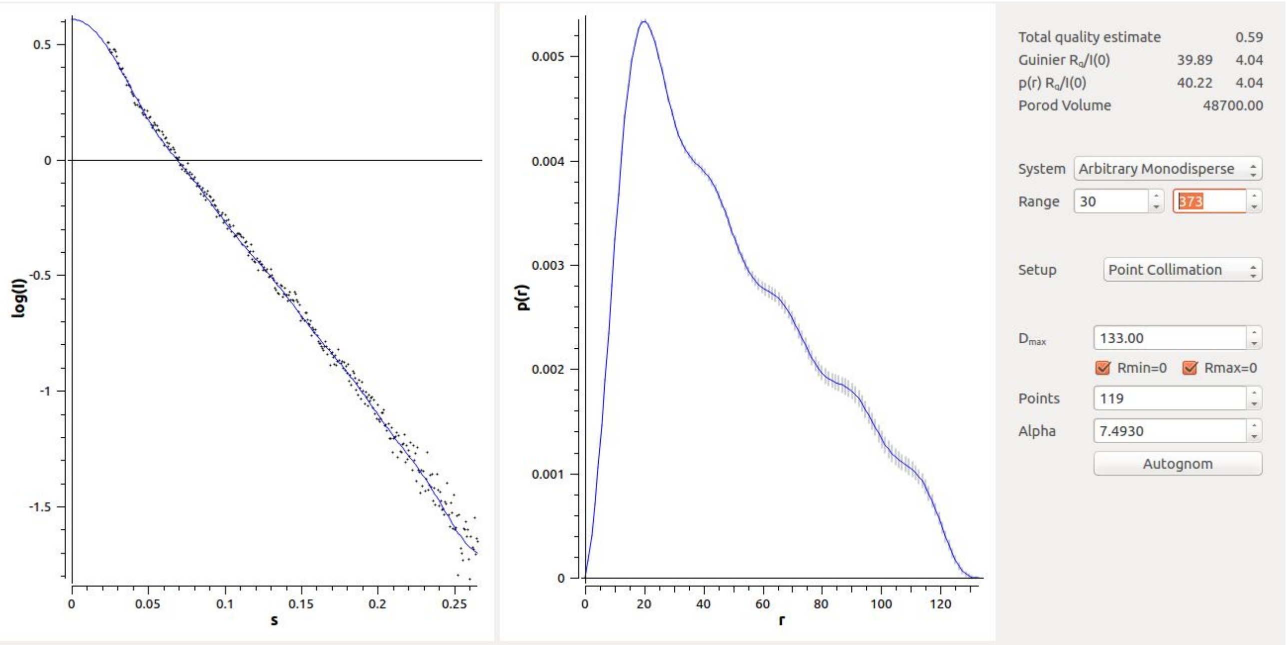

Figure S9. Fit of the 1D-folded SAXS pattern from 50 mg/ml solutions of clusters in hexane, returning a 4.0±0.4 nm gyration radius for the cluster, and clearly showing an asymmetric pair distribution function p(r) peaked at about 2.5 nm and featuring a maximum inter-atomic distance (i.e. cluster long axis) of about 13.3 nm (reported numbers for the scattering vector (s) and the direct space vector ® are in $Å^{-1}$ and Å units, respectively).

The NCL geometrical parameters determined by SAXS measurements are compatible with those determined by PDF. The maximum interatomic distance D_{max} resulting from SAXS measurements is compatible with the NCL equatorial length of 13 nm estimated by PDF. In addition, the SAXS determination of the radius of gyration (4.0±0.4 nm) is in agreement with PDF determinations of the NCL shape. In fact, the radius of gyration for an oblate ellipsoid having equatorial and polar radii respectively Erad and Prad is given by[4]:

$$R_g = \frac{8}{5}E_{rad}^{\ 2} + \frac{E_{rad} + 4P_{rad}}{E_{rad} + 2P_{rad}}.$$

Thus, if we consider the experimental values determined by PDF: Erad=6 nm and Prad=0.8 nm we expect Rg=3.9 nm.

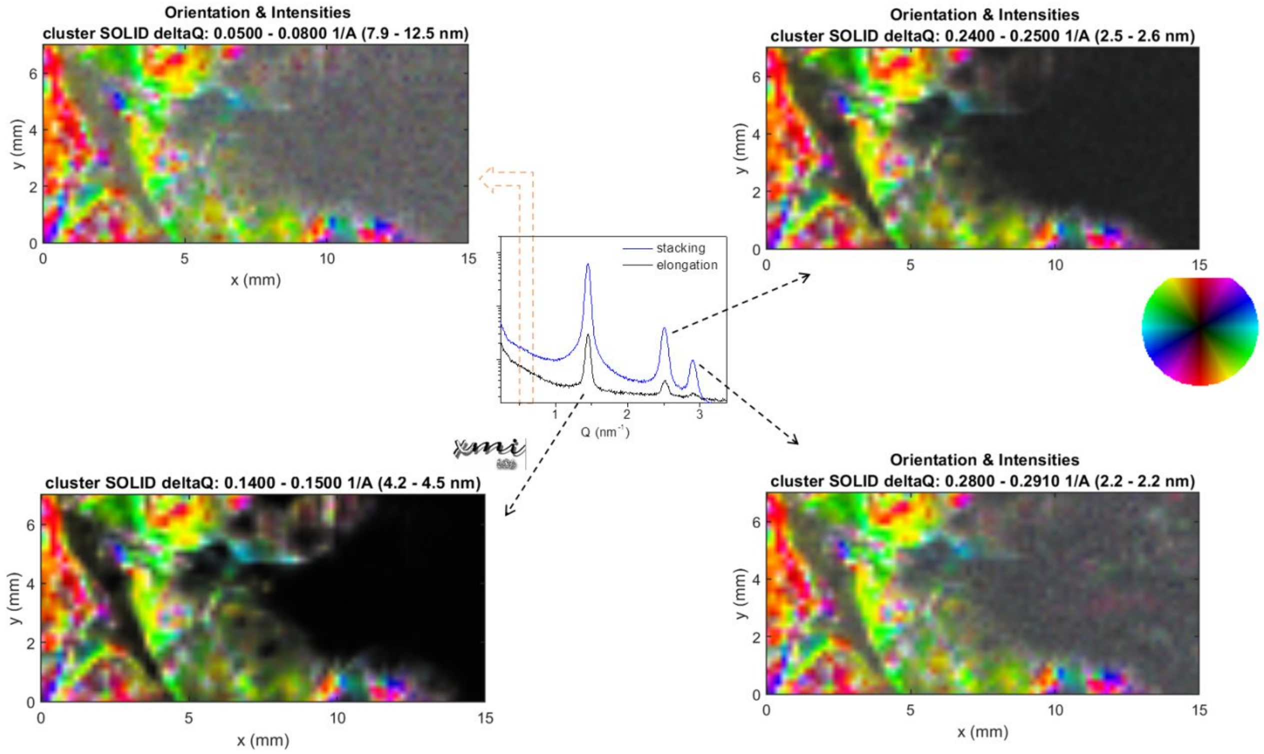

Figure S10. Orientation/Intensity SAXS microscopies for each of the three peaks detected in the SAXS pattern, and for the low-Q range diffuse scattering related to the particle form factor. The preferred azimuthal orientation of the scattering and the relevant direction is described by the local colour, based on the colour wheel on the right side of the figure. Colour brightness is proportional to the scattering intensity.

The orientation of the nanoscale ordered domains at any sample position is indicated in Fig. S8 by a well-defined color, according to the color wheel on the right side of the Figure. SAXS microscopies show that all partial rings in the SAXS pattern are always mutually aligned, although their overall orientation can change across the investigated sample area. The orientation distribution of the diffuse scattering appearing at small Q-values is also plotted in the top left corner of Fig. S8, and shows to be coherent (similar distribution of colors) with the orientation distribution obtained for the three diffraction peaks. Since diffuse scattering can be ascribed to the form factor of the NCLs, this indicates that the spheroids are oriented with their short and long axes respectively parallel and perpendicular to the stacking direction.

---

[4] John Satterly, Formulae for Volumes, Surface Areas and Radii of Gyration of Spheres, Ellipsoids and Spheroids The Mathematical Gazette 44, 347 (1960), 15-19.
